# Mechanistic Insights into Vegetable Color Stability: Discoloration Pathways and Emerging Protective Strategies

**DOI:** 10.3390/foods14132222

**Published:** 2025-06-24

**Authors:** Jianing Zhang, Junjun Zhang, Lidan Zhang, Yuhong Xue, Ke Zhang

**Affiliations:** Agricultural Product Processing and Storage Lab, School of Food and Biological Engineering, Jiangsu University, Zhenjiang 212013, China; zjn983615@163.com (J.Z.); 19710510382@163.com (L.Z.); xue_yuhong@163.com (Y.X.); 18253753979@163.com (K.Z.)

**Keywords:** vegetables, color protection, enzymatic browning, non-enzymatic browning, chlorophyll degradation, discoloration mechanism

## Abstract

During processing and storage, vegetables often experience undesirable color changes, including fading, lightening, or yellowing and softening, due to browning (enzymatic and non-enzymatic) and chlorophyll degradation. These changes diminish commercial and nutritional value. Therefore, it is necessary to maintain vegetable color and improve the quality of vegetable-based dishes. To address these issues, it is a scientific and practical necessity to summarize and discuss existing strategies and innovative techniques. This review first highlights the mechanisms of vegetable browning. This review then provides a comprehensive overview of recent advances in methods for color preservation, focusing on underlying mechanisms and techniques for inhibiting color changes from physical, chemical, and biological perspectives. A review of innovative technologies suggests that effective color preservation in vegetables is achieved by inhibiting the conditions that lead to three unfavorable color change reactions: enzymatic browning, non-enzymatic browning, and chlorophyll degradation. Current research frequently employs combined approaches that integrate two or more techniques to mitigate these adverse color changes. Moreover, most of these methods could simultaneously inhibit the three reaction processes. Future research directions are proposed for in-depth investigations into the molecular mechanisms of color changes in vegetables and the impact of treatments on the nutritional value.

## 1. Introduction

Each year, over 30% of harvested vegetables are discarded globally due to color degradation, resulting in economic losses exceeding USD 50 billion and diminishing nutritional quality [1]. Color deterioration not only reduces consumer appeal but also serves as a visible marker of underlying biochemical spoilage that may compromise food safety [2,3]. As color is the first visual cue consumers use to assess freshness and quality, discoloration severely limits the commercial value and shelf-life of vegetable products.

Vegetables especially play a vital role in such diets due to their rich nutritional profiles and health-promoting properties [4]. According to the Food and Agriculture Organization of the United Nations (FAO), vegetables supply more than 90% of the daily required intake of vitamin C and over 65% of vitamin A for the human body [5]. Additionally, vegetables are rich in phenolic compounds, dietary fiber, organic acids, and bioactive sulfur-containing compounds such as dipropylene and methyl sulfide compounds, which contribute to enhance immune function and the prevention of chronic diseases [6]. Consequently, ensuring the quality and availability of vegetables is critical to addressing global nutritional deficiencies and advancing public health.

However, vegetables are highly perishable and especially vulnerable to spoilage during post-harvest handling, storage, and distribution [7]. Among all quality attributes, color is the most rapidly affected and strongly influences consumer purchasing decisions [8]. Discoloration—particularly browning and chlorophyll degradation—has been identified as a major cause of rejection in the global vegetable market, leading to substantial economic losses [9,10].

**Figure 1:**
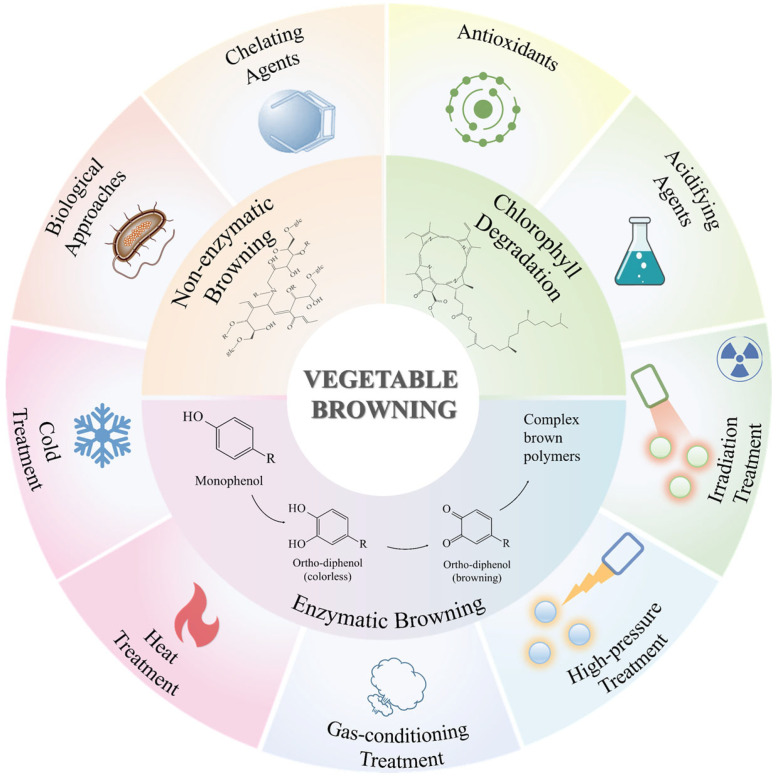
The Browning mechanism and protection mechanism of vegetables.

Although various reviews have examined enzymatic browning and its inhibition strategies, there is a lack of comprehensive analyses that integrate all forms of discoloration—including non-enzymatic browning and chlorophyll degradation—with emerging control methods. Furthermore, existing literature tends to focus either on degradation mechanisms or on individual preservation strategies, without bridging the two perspectives. In this review, we address this gap by systematically connecting the molecular mechanisms behind vegetable discoloration with the most recent advances in color stabilization. We synthesize studies published between 2020 and 2025, focusing on three major intervention approaches: physical, chemical, and natural anti-browning strategies. By presenting an integrated overview, this review offers an actionable framework for improving vegetable shelf-life, reducing waste, and promoting the commercialization of visually appealing, nutritious plant-based products. The content of the review structure is shown in Figure 1.

Relevant studies were retrieved from the Web of Science and PubMed databases (2020–2025) using keywords such as ‘vegetable color stability’, ‘pigment degradation’, and ‘protective interventions’. We applied inclusion criteria of English-language original research articles and comprehensive reviews that reported quantitative data or mechanistic insights into vegetable pigment stability. Exclusion criteria included conference abstracts, book chapters, patents, non-English studies, and irrelevant topics. A total of 512 records were initially identified, of which 189 met the eligibility criteria and were included in this review.

## 2. Mechanism of Vegetable Color Change

There are two main factors for color changes in vegetable-based products: browning and chlorophyll degradation. Browning is categorized into enzymatic and non-enzymatic types [9]. Among them, enzymatic browning significantly influences the color change in vegetable-based products.

### 2.1. Enzymatic Browning

#### 2.1.1. Mechanism of Enzymatic Browning

Enzymatic browning is a complex biochemical process that is usually described by the theory of the regionalized distribution of phenolic compounds in plant tissues and the enzymes that induce their reactions [11]. According to this theory, the spatial separation of these components plays a critical role in preventing undesirable browning reactions. Phenolic compounds, the primary substrates of enzymatic browning, are typically sequestered in specific intracellular vesicles, while polyphenol oxidase (PPO), the enzyme responsible for their oxidation, is located in distinct cytoplasmic compartments [12]. This compartmentalization ensures that phenolics and PPO remain separated under normal physiological conditions, thereby inhibiting browning reactions despite the presence of oxygen, a key reactant in the process [13].

The cell is a highly organized structure with organelles surrounded by a selectively permeable film, which helps delineate the intracellular environment. In intact cells, this organization provides protection against the enzymatic browning reaction despite the presence of oxygen (a key component of enzymatic browning). However, compartmentalization is disrupted when the cell wall is physically damaged, mechanically damaged, or damaged by enzymatic breakdown. This allows phenolic compounds that were in the vesicles to come into direct contact with PPO and oxygen in the cytoplasm, triggering a series of biochemical reactions. Specifically, these reactions are the oxidation of phenolic substrates. The phenolic substrate is polymerized into larger, more complex structures during the reaction. The resulting melanoidins then lead, at a macroscopic level, to the dark brown discoloration observed in damaged or cut vegetable tissues [14].

The formation of melanoidins is often associated with the degradation of antioxidants and other essential nutrients, thereby not only affecting the visual quality of vegetables but also diminishing their nutritional value and shelf-life [15]. The rate and extent of enzymatic browning are influenced by several factors, including temperature, oxygen availability, and PPO activity, making its control and inhibition particularly challenging.

#### 2.1.2. Conditions of Enzymatic Browning

**Figure 2:**
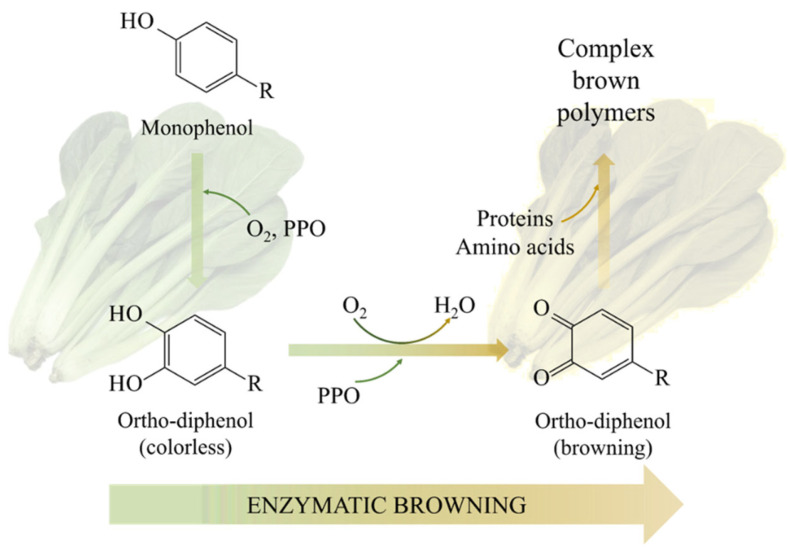
Mechanism of enzymatic browning.

Enzymatic browning is a chemical reaction involving several interrelated processes. As shown in Figure 2, these chemical reactions proceed sequentially leading to characteristic browning of plant tissues. First, monophenols are oxidized to o-diphenols by PPO in the presence of oxygen. This initial reaction forms colorless o-diphenol, which serves as the main substrate for further oxidation. These o-diphenols are further oxidized to form o-quinone under the catalytic action of PPO. O-quinone is a highly reactive intermediate. Immediately thereafter, o-quinones through interactions with proteins, amino acids and other cellular components. Complex brown polymers are formed which ultimately lead to visible browning of plant tissues [14].

The enzymatic browning reaction is continuous. This means that the reaction continues as long as reducing substances, such as phenolic compounds and sufficient enzyme activity, are still present. Once these substrates are depleted or the enzyme activity is reduced due to various factors, the reaction slows down and eventually stops [16]. The enzymatic browning process fundamentally depends on the presence of three critical components: oxygen, PPO, and phenolic substrates [17]. All three must be simultaneously present for browning to proceed efficiently. Consequently, effective control of these factors is key to minimizing enzymatic browning, which is particularly crucial in the post-harvest preservation of vegetables [18].

In addition to these primary factors, enzymatic browning is also affected by a number of secondary environmental conditions such as temperature, light and pressure. High temperatures can provide more kinetic energy to increase the rate of chemical reactions. On the contrary, low temperatures can slow down the activity of PPO, which in turn reduces the browning process [19]. Light exposure has been reported to enhance reactive oxygen species production, which can increase PPO activity and thus promote browning [20]. In addition, external pressure affects enzyme conformation and activity, and high pressure may inhibit PPO activity or alter its substrate specificity [21].

Thus, while oxygen, enzymes, and phenolics are necessary for enzymatic browning, external conditions such as temperature, light, and pressure are secondary factors that influence the enzymatic reaction [22]. A comprehensive understanding of the interactions between these primary and secondary factors is essential for developing effective strategies to mitigate enzymatic browning in fresh produce.

### 2.2. Non-Enzymatic Browning

#### 2.2.1. Mechanism of Non-Enzymatic Browning

Non-enzymatic browning is also a key factor in the color stability, quality and shelf-life of vegetable products. Unlike enzymatic browning, non-enzymatic browning occurs through a number of different chemical reactions. These chemical reactions do not require enzymes, but still result in undesired color changes and quality deterioration. Previous research has identified several reactions that fall under the category of non-enzymatic browning, including the Maillard reaction, caramelization reaction, degradation of vitamin C, and polyphenol oxidative condensation [23]. The Maillard reaction is the most widely studied and the most important contributor to non-enzymatic browning of foods, including vegetables.

The Maillard reaction involves a complex sequence of interactions between reducing sugars and amino compounds, typically amino acids or proteins. Initially, the carbonyl group of a reducing sugar (e.g., glucose or fructose) reacts with the amino group of an amino compound to form an unstable Schiff base. This intermediate rapidly undergoes an Amadori rearrangement to yield more stable Amadori products, which are highly reactive. Under various environmental conditions, these intermediates degrade into a range of compounds, including unsaturated aldehydes with strong browning potential. Ultimately, these reactions lead to the formation of high-molecular-weight melanoidins, which are responsible for the characteristic browning observed in processed and stored foods [24,25].

Current research has focused on the extensive study and characterization of the formation of the intermediate products of the Maillard reaction, and the mechanism of the production of the final high-molecular-weight polymer is still poorly understood. Therefore, recent studies have focused on the effect of various processing conditions (e.g., temperature, moisture content, and pH value) on the formation of the final product melanoidin. As with enzymatic browning, these high-molecular-weight products not only lead to browning, but also alter the flavor, texture, and nutritional composition of the food. In addition, the Maillard reaction interacts with other non-enzymatic browning reactions (e.g., caramelization and polyphenol oxidative condensation), further complicating the overall non-enzymatic browning process. Understanding the whole process of non-enzymatic browning reactions and their underlying mechanisms, especially the formation of high-molecular-weight melanoidins, is essential for developing strategies to control and mitigate browning of vegetable products in order to improve their color stability and extend their shelf-life.

#### 2.2.2. Conditions of Non-Enzymatic Browning

The mechanism of non-enzymatic browning is complex and involves many chemical reactions that produce a large number of intermediates. These reaction processes are influenced by a variety of factors and are difficult to control completely. Therefore, effective control and inhibition of non-enzymatic browning can only be achieved by looking at the external conditions that affect the chemical reactions, such as temperature, time, pH and the presence of specific precursor compounds. Previous study has found that the complexity of non-enzymatic browning stems from its multi-step nature. These reactions are sensitive to environmental conditions external to the reaction, which in turn significantly affects the rate and extent of browning in different foods [26].

For example, Sacchetti et al. reported that high-temperature, short-time treatments led to less non-enzymatic browning in cocoa beans compared to prolonged heating, indicating that the balance between temperature and time is crucial in controlling browning intensity. Their study also highlighted that extended cooking or overheating can accelerate the Maillard reaction and other non-enzymatic pathways, resulting in excessive pigment formation and deterioration of flavor and nutritional quality [27]. In addition, Liu et al. found that pH plays a vital role in modulating browning; lower pH values were shown to inhibit the formation of reactive intermediates, thereby reducing the generation of high-molecular-weight browning compounds. This suggests that pH adjustment during food processing or storage may be an effective strategy to suppress non-enzymatic browning in vegetables and other products [28].

In addition to processing conditions, the chemical composition of a food also plays a key role in determining the rate of non-enzymatic Browning. It has been found that ascorbic acid, fructose, and arginine can act as pre-reactive agents of non-enzymatic Browning, accelerating or altering the process of non-enzymatic Browning [29]. Different amino acids have different interactions with reducing sugars and have different effects on non-enzymatic Browning. For example, basic amino acids, such as lysine and arginine, tend to form more reactive intermediates with sugars, which can lead to faster Browning. In contrast, non-polar or neutral amino acids may produce slower Browning rates due to their chemical properties [30]. The structure of sugars also plays a role—simple sugars such as glucose and fructose are more susceptible to browning than complex carbohydrates due to their higher reactivity in Maillard-type reactions [31].

At the same time, other conditions can also affect the non-enzymatic Browning process. Guo et al. found that the presence of certain metal ions, such as calcium and magnesium, may be involved in active intermediates or enzymatic interactions of Browning, preventing the formation of unwanted pigments and thereby inhibiting the Maillard reaction [32]. Grant-Preece et al. have shown that exposure to light may speed up or slow down the rate of non-enzymatic Browning, depending on the specific wavelength and intensity of the light [33]. Shishir et al. found that modified atmosphere packaging could inhibit browning by controlling oxygen levels or incorporating light-blocking materials [34].

In summary, non-enzymatic Browning is a highly complex process that depends on a range of factors, including temperature, time, pH, and the chemical composition of the food. While certain conditions can accelerate Browning, other conditions, such as acidic pH or the presence of metal ions, can help slow it down. Understanding these factors and their interactions is critical to developing strategies to control non-enzymatic Browning.

### 2.3. Chlorophyll Degradation

#### 2.3.1. Mechanism of Chlorophyll Degradation

In green vegetables, the degradation of chlorophyll is a key factor leading to color loss and significantly affects the overall quality of the product, including its visual appeal, nutritional content and consumer acceptability [35]. Chlorophyll refers to the green pigment responsible for capturing light energy during photosynthesis. It exists mainly in two forms: chlorophyll-a and chlorophyll-b. The two types of chlorophyll differ in their chemical structure. Chlorophyll-a has a methyl group (-CH_3_) on the II pyrrole ring, while chlorophyll-b has an aldehyde group (-CHO) in the same location on the pyrrole ring [36]. Both forms of chlorophyll play a crucial role in photosynthesis. Part of this chlorophyll-a is the main pigment in the light harvesting process, while chlorophyll-b assists in capturing light energy and transferring it to chlorophyll-a. In the process of green vegetable storage, due to temperature, light, pH value, mechanical damage and other environmental factors will cause chlorophyll degradation, which leads to adverse color reactions in vegetables.

The degradation of chlorophyll is catalyzed by multiple chemical reactions. First, the chlorophyll enzyme catalyzes the shedding of the phthalein group in chlorophyll-a, converting it into demethyl-chlorophyll-a and chlorophyll-alcohol. This makes the pigment less stable and more prone to further degradation. Subsequently, de-magnesium chelatase catalyzes the removal of magnesium ions in chlorophyll-a to produce de-magnesium chlorophyll-a, which is a key step in chlorophyll degradation. The stability of pheophyll-a is significantly lower than that of chlorophyll-A, making it more susceptible to oxidation and further transformation. Then, pheophyll-a is further enzymolized by pheophyllase oxygenase and red chlorophyll metabolite reductase, resulting in the formation of fluorescent chlorophyll metabolites. As a result, green pigments are gradually replaced by other decomposition products, producing brown or yellow color changes [37,38].

#### 2.3.2. Conditions of Chlorophyll Degradation

Chlorophyll enzymes play a central role in the breakdown of chlorophyll molecules during post-harvest storage and food processing. However, studies have shown that the relationship between chlorophyll enzyme activity and chlorophyll degradation rate is not linear, suggesting that various factors may affect enzyme activity in complex ways [39]. Therefore, any factor that directly or indirectly affects the activity of chlorophyll enzymes will have an impact on the chlorophyll degradation rate.

For instance, acidic environments have been reported to inhibit chlorophyllase activity by altering its structure and reducing its catalytic efficiency, thereby slowing chlorophyll breakdown [40]. Therefore, protecting the color of green vegetables during processing and controlling pH is a key step. Especially in curing or pickling operations, acidifiers are often added to preserve color and extend shelf-life. In addition, light is another key factor affecting chlorophyll degradation. Zhang et al. showed that light can induce ROS production, promote oxidation reaction and accelerate the decomposition of chlorophyll in plant tissues [41]. Reducing light during storage or processing is a key strategy to maintain chlorophyll content and color stability in vegetables. Therefore, it is often recommended to use light-blocking packaging materials or store in dark conditions to extend the shelf-life of green vegetables.

Temperature also significantly impacts chlorophyll degradation. Elevated temperatures can enhance enzyme activity, including that of chlorophyll-degrading enzymes, thereby accelerating chlorophyll breakdown and resulting in rapid color loss [42]. However, temperature interacts with other environmental variables, such as oxygen availability and humidity, to collectively determine the degradation rate. Proper control of temperature and humidity during storage and transportation is therefore essential to preserving vegetable color.

Metallic elements can also interact with chlorophyll molecules, catalyzing or inhibiting various degradation pathways. Yang et al. found that certain metals, such as iron and copper, can act as catalysts during oxidation, promoting the breakdown of chlorophyll. In contrast, magnesium, as the central atom in the chlorophyll molecule, can play a stabilizing role by binding to the chlorophyll structure and maintaining its integrity [43]. It is worth noting that oxygen levels also play a crucial role in the chlorophyll degradation process. Studies have shown that low-oxygen environments can effectively inhibit chlorophyll degradation even if they do not directly affect chlorophyll enzyme activity [44]. Therefore, controlling the oxygen environment during storage and packaging may be an effective way to both maintain the green color of vegetables and minimize the loss of valuable nutrients.

In short, the preservation of chlorophyll in vegetable products is a complex process, which is affected by many factors. Strategies such as controlling pH, limiting light, optimizing temperature conditions and using air-conditioned packaging help to slow chlorophyll degradation and ensure that the vegetable’s color and nutrients are preserved throughout its shelf-life. These approaches are critical to both consumer satisfaction and the economic viability of the fresh produce industry [45].

## 3. Methods to Inhibit Browning

Adverse color changes in vegetables during processing and storage not only diminish market appeal but also lead to nutritional loss and may lead to the formation of harmful compounds [10]. To address the issue, several strategies have been employed, including physical, chemical, and natural anti-browning method. The inhibition methods typically target and mitigate the three factors of color deterioration on varying degrees, thereby exhibiting a multifaceted effect.

### 3.1. Physical Methods

#### 3.1.1. Cold Treatment

Cold treatment remains a cornerstone technology for inhibiting enzymatic and non-enzymatic browning in fresh-cut vegetables, primarily through metabolic suppression and enzyme inactivation. However, conventional freezing methods often compromise cellular integrity due to the formation of macro-scale ice crystals, which mechanically disrupt cell walls and accelerate pigment degradation [46]. This structural damage creates a paradoxical effect where the initial preservation method exacerbates quality deterioration through two pathways: (1) facilitating contact between phenolic substrates and PPO, and (2) promoting ascorbic acid oxidation through cellular compartmentalization loss [10,47]. Recent technological advancements attempt to resolve this dilemma through ice crystal morphology control. Notably, researchers demonstrated that high-pressure carbon dioxide freezing (HPCF) generates smaller, more uniform ice crystals in lettuce, achieving 53.50% lower polyphenol oxidase activity and 74.63% lower total phenolic content [48]. While these results are promising, the industrial scalability of HPCF warrants critical evaluation due to its high equipment costs and energy demands [49].

Emerging evidence suggests that synergistic approaches combining physical and biochemical interventions may offer more practical solutions. Isochoric freezing, which maintains constant volume during phase change, has shown particular effectiveness in preserving chlorophyll and ascorbic acid in leafy greens compared to isobaric systems [50]. This method, characterized by its capacity to maintain uniform temperature distribution and minimize ice crystal damage, has demonstrated significant advantages across different vegetable types. The schematic of isochoric freezing is shown in Figure S1. For instance, in spinach, thawed samples subjected to isometric freezing retained cellular integrity and exhibited levels of cell lysis comparable to fresh counterparts, indicating effective structural preservation [49]. In the case of potato, this technique not only delayed enzymatic browning by approximately one week but also enhanced the total phenolic content and antioxidant capacity of the samples, suggesting that isometric freezing may induce beneficial stress responses while inhibiting undesirable quality degradation [51]. These findings underscore the dual benefits of isometric freezing in maintaining both the physicochemical stability and nutritional functionality of vegetables during storage.

Future research should prioritize three key areas: (1) developing energy-efficient phase change materials for sustainable cold chains, (2) establishing vegetable-specific freezing protocols considering cellular architecture differences, and (3) integrating real-time quality monitoring systems with adaptive preservation parameter adjustment. Recent advances in hyperspectral imaging for early browning detection [52] and AI-driven predictive models for shelf-life estimation [53] provide critical enabling technologies for these developments.

#### 3.1.2. Heat Treatment

Heat treatment has emerged as a dual-functional intervention for fresh-cut vegetable preservation, achieving enzymatic inactivation through both thermal denaturation of PPO and strategic modulation of cellular compartmentalization [54,55]. While conventional hot water blanching at 80–100 °C can reduce PPO activity by 60–80%, it simultaneously disrupts pectin-cellulose networks, causing texture softening and creating aqueous microenvironments that paradoxically enhance microbial proliferation [56]. This fundamental limitation has spurred the development of precision thermal technologies that decouple enzyme inactivation from structural degradation.

High-humidity hot air impingement blanching (HHAIB) has emerged as an effective thermal pretreatment for preserving the post-harvest texture and visual quality of vegetables. Specifically, HHAIB treatment at 110 °C with 40% relative humidity for 38 s has been shown to maintain the crispness of chili peppers while achieving minimal POD residual activity (0.52%) and a low browning index difference (7.09) [57]. These effects are primarily attributed to the rapid vapor condensation on the vegetable surface, which generates a protective thermal boundary layer that prevents excessive moisture loss and structural collapse. By limiting enzymatic activity and preserving cellular integrity, HHAIB offers a promising approach for extending the shelf-life and sensory attributes of fresh produce with minimal thermal damage [58]. The heat transfer mechanism description of HHAIB was shown in Figure S2.

Similarly, microwave-assisted blanching (MAB) at 300 W offers a time-efficient and nutrient-conserving alternative to conventional water blanching. By leveraging selective dielectric heating, MAB preferentially targets aqueous regions rich in PPO, thus accelerating enzyme inactivation. As a result, this method enhances ascorbic acid retention (13.59 mg/100 g), outperforming traditional water blanching (10.24 mg/100 g), and simultaneously reduces thermal exposure time [59]. However, these technologies face critical scalability challenges. HHHAS requires complex humidity control systems increasing capital costs by 30%, while microwave heterogeneity causes uneven treatment in dense vegetable tissues [60].

Nevertheless, critical knowledge gaps persist: (1) Nutrient–thermal stability trade-offs: While vitamin C degradation follows first-order kinetics, recent metabolomics reveals unexpected flavonoid enhancement through stress-induced biosynthesis [61]. (2) Matrix-specific thermal tolerance: Cruciferous vegetables exhibit higher heat resistance than leafy greens due to glucosinolate-mediated membrane stabilization, necessitating species-specific protocols [62]. Future research should prioritize intelligent thermal systems incorporating real-time spectroscopy feedback and adaptive control algorithms. The integration of pulsed electric fields with mild heating presents promise, with preliminary data showing that at reduced heat loads, PEFs inhibit browning by suppressing respiration rates during storage, and minimize nutrient loss from food by maintaining structural integrity [63,64]. Such hybrid approaches may ultimately reconcile the conflicting demands of microbial safety, nutritional preservation, and environmental sustainability in fresh-cut vegetable processing.

#### 3.1.3. Gas-Conditioning Treatment

Gas-conditioning technologies, particularly modified atmosphere packaging (MAP), have evolved beyond simple gas composition adjustments to become sophisticated systems that dynamically interact with the physiological and biochemical pathways of fresh-cut vegetables [11]. The functional mechanism is illustrated in Figure S3. While traditional MAP can reduce browning, its fixed gas permeability often fails to accommodate the fluctuating respiration rates of high-metabolic produce, leading to anaerobic fermentation and the accumulation of off-flavors [65,66]. Although recent advancements in gas-responsive packaging have addressed some of these preservation challenges, a fundamental tension remains between technical performance and practical scalability.

Emerging systems employing pH-activated nanocomposite membranes exhibit enhanced gas modulation, with certain moisture-sensitive designs enabling CO_2_ enrichment while simultaneously inhibiting spoilage [67]. These adaptive materials offer promising gas selectivity and have demonstrated efficacy in reducing microbial populations and enzymatic browning. However, practical implementation remains difficult, especially with high-respiration produce. In leafy vegetables, excessive moisture exchange can paradoxically induce hypercapnic conditions, triggering secondary metabolic shifts such as alcohol accumulation and quality degradation. This challenge underscores the need for next-generation materials that can adapt dynamically to environmental changes, balancing gas composition and humidity management [68].

Concurrently, noble gas blends redefine inert gas roles by quenching singlet oxygen and stabilizing membranes, reducing lipid peroxidation by 50% and an effective reduction in microbial load [69]. Emerging 3D-MAP system integrates spatiotemporal control with 3D-printed gradient films and β-CD inclusions, but sustainability trade-offs remain: reduced carbon footprint but lack of mechanical robustness in bio-based films [70]. Future advancements must bridge nanoscale interventions with systems biology and digital twins, transitioning from empirical gas recipes to precision-controlled “gas-microbiome-vegetable” ecosystems that harmonize preservation efficacy, nutritional integrity, and environmental sustainability.

#### 3.1.4. High-Pressure Treatment

High-pressure processing (HPP) has emerged as a dual-functional technology for fresh-cut vegetable preservation, leveraging hydrostatic pressure (typically 100–600 MPa) to simultaneously inhibit enzymatic browning and microbial proliferation while modulating cellular water dynamics [71]. By creating a transient pressure gradient across vegetable tissues, HPP restricts intracellular water migration, reducing dehydration-induced mass loss and preserving ascorbic acid through physical suppression of oxidative reactions, as demonstrated in pressurized argon-treated potatoes [72].

The technology’s ability to downregulate ethylene biosynthesis and respiratory rates extends shelf-life by delaying senescence, while its non-thermal nature minimizes thermal degradation of heat-sensitive nutrients [73]. However, the purported “sterilization-preservation synergy” remains contentious: although HPP achieves reductions in microorganisms, its sublethal stress (200–400 MPa) may induce microbial stress responses, enhancing biofilm formation and antibiotic resistance in surviving populations—a critical food safety paradox requiring mechanistic resolution [74]. Furthermore, the narrow therapeutic window between effective preservation and cellular damage poses significant challenges, with excessive pressure disrupting chloroplast ultrastructure and plasmalemma integrity, leading to irreversible texture softening and chlorophyll leakage. Current limitations in scalability and species-specific optimization—rooted in variable cellular architecture (e.g., parenchyma-rich vs. lignified tissues)—hinder broad adoption, as evidenced by inconsistent outcomes between leafy greens and root vegetables under identical protocols.

Future advancements must bridge fundamental research and industrial pragmatism through: (1) multiscale modeling of pressure-vegetable-microbiome interactions to define species-specific pressure thresholds, (2) hybrid systems integrating HPP with edible coatings to mitigate sublethal microbial recovery, and (3) energy-efficient pressure cycling technologies that minimize structural damage while enhancing process uniformity. By addressing these interdisciplinary challenges, HPP could transition from a niche intervention to a cornerstone of sustainable post-harvest strategies, balancing microbial safety with organoleptic and nutritional integrity.

#### 3.1.5. Irradiation Treatment

Irradiation technologies, particularly ultraviolet (UV-C at 254 nm) and blue light (450 nm), have demonstrated dual functionality in fresh-cut vegetable preservation by simultaneously targeting microbial inactivation and modulating enzymatic browning pathways [75]. The bactericidal action of UV irradiation primarily arises from the formation of thymine dimers in microbial DNA, which disrupt replication and lead to cell death. Meanwhile, blue light exerts selective inhibitory effects on PPO isoforms via flavin-mediated photochemical reactions, as demonstrated by Zhao et al., who reported a significant reduction in browning indices of treated yam samples [76]. However, the practical effectiveness of UV-based interventions is hampered by their limited penetration depth. Surface moisture can scatter up to 70% of UV photons, creating protective niches for microbes within epidermal crevices—a limitation further exacerbated by the irregular and heterogeneous surface morphologies of many vegetables [77]. While combined UV-blue light strategies offer enhanced efficacy, they may also induce oxidative stress in plant tissues, accelerating ascorbic acid degradation and promoting membrane lipid peroxidation [78]. Emerging solutions like pulsed light [79] and plasma-activated UV systems [80] demonstrate improved uniformity, yet their scalability remains constrained by energy inefficiency and photoreactivation risks.

The broader landscape of physical preservation methods—from irradiation to high-pressure processing—faces a universal paradox: interventions suppressing browning often inadvertently compromise nutritional or textural integrity. For instance, gamma irradiation (3 kGy) reduces Bamboo shoot PPO activity but degrades nutrient composition through radiolytic cleavage [81]. Future innovations must prioritize intelligent synergy, such as UV-adaptive packaging with light-diffusing nanostructures to enhance irradiation uniformity, or hybrid systems coupling e-beam irradiation with antioxidant-releasing edible films to offset oxidative collateral damage. Table 1 provides an overview of recent innovations in the application of physical methods to inhibit vegetable Browning, showing the various advances and their respective results.

### 3.2. Chemical Methods

#### 3.2.1. Acidifying Agents

Acidification approaches in fresh-cut produce preservation operate through pH-mediated enzyme suppression, capitalizing on the inverse correlation between acidic environments and oxidative enzyme functionality. Browning reactions driven by PPO and POD exhibit maximum activity in neutral pH conditions where specific metal ion coordination within the enzyme’s catalytic site becomes optimal. By strategically lowering pH to mildly acidic ranges, common organic acid treatments disrupt critical amino acid residues essential for enzymatic catalysis, particularly through protonation mechanisms affecting metal-binding domains. This pH-dependent interference leads to substantial reduction in oxidative enzyme performance, effectively decelerating pigment-generating biochemical pathways [121]. This pH manipulation concurrently disrupts non-enzymatic browning pathways: acidic environments inhibit Maillard reactions by protonating amino groups (-NH_2_ → -NH_3_^+^), effectively blocking Schiff base formation between reducing sugars and amino acids [28]. However, the technology’s purported dual antimicrobial–antioxidant benefits mask critical trade-offs—low pH induces proton stress in plant cells, accelerating chlorophyll degradation via Mg^2^^+^ displacement from porphyrin rings and triggering up to anthocyanin loss in pigmented vegetables (carrots, red cabbage), paradoxically diminishing visual appeal despite reduced browning [122].

Current limitations stem from oversimplified application paradigms. While citric and ascorbic acids remain industry standards, their buffering capacity varies dramatically. Repeated applications must therefore be made or there is a risk of sensory fatigue (acidity greater than the acceptability threshold of 2.5%) [123]. Future innovation must address the spatial heterogeneity of acid diffusion in vegetable tissues. Hybrid approaches integrating pH-triggered nanoemulsions (e.g., alginate-coated malic acid microparticles) with predictive AI-driven dosing systems could resolve these gradients, enabling precision acidification tailored to cellular architecture and real-time metabolic feedback. Such advancements would transform acidification from a blunt empirical tool to a dynamic, tissue-specific preservation modality.

#### 3.2.2. Antioxidants

Antioxidants, while pivotal in mitigating enzymatic browning through redox modulation of quinones and stabilization of phenolic intermediates, face context-dependent efficacy challenges that demand critical reappraisal. Ascorbic acid, the industry benchmark, suppresses browning via a dual mechanism: chemically reducing o-quinones and competitively binding to PPO active sites [124]. However, its non-enzymatic regeneration cycle is pH- and light-sensitive—under fluorescent storage lighting, ascorbate degradation accelerates by 40% at pH > 5.5, paradoxically increasing semiquinone radical accumulation and subsequent browning rebound [125]. This temporal instability highlights a critical gap in single-antioxidant strategies, exacerbated by interspecies variability: cruciferous vegetables are more ascorbate stability than leafy greens due to glucosinolate-mediated redox buffering [126]. Emerging alternatives like melatonin present a paradigm shift, leveraging pleiotropic mechanisms beyond mere radical scavenging. At 100 μM, melatonin not only quenches peroxyl radicals (k = 2.1 × 10^6^ M^−1^s^−1^) but also upregulates glutathione biosynthesis by 50% through NPR1-mediated salicylic acid pathways, as evidenced in *Agaricus bisporus* [127].

However, melatonin’s hydrophobic nature limits its solubility and diffusion in aqueous tissues, necessitating advanced delivery systems such as nanoemulsion encapsulation to improve bioavailability and tissue penetration [128]. The field now confronts a trilemma: balancing antioxidant efficacy, economic viability, and sensory compatibility. Future innovation must prioritize smart delivery systems—pH-responsive hydrogels or light-activated nanozymes—that spatially and temporally control antioxidant release, aligning redox activity with vegetable metabolic rhythms to achieve precision preservation without compromising affordability or sustainability.

#### 3.2.3. Chelating Agents

Chelators, while effective in disrupting metal-catalyzed browning pathways, present a complex interplay between targeted inhibition and unintended metabolic consequences that demand nuanced evaluation. By sequestering Cu^2^^+^ from PPO active sites, conventional chelators like EDTA reduce enzymatic browning in model systems [129]. However, their performance in real food matrices is highly variable and species dependent. For example, cruciferous vegetables exhibit significantly higher resistance to PPO-metal chelation than leafy greens, largely due to glucosinolate-mediated shielding of metal ions [130,131]. Non-enzymatic preservation strategies employing metal chelation demonstrate dualistic metabolic impacts in plant tissues. While iron-binding approaches effectively inhibit lipid oxidation processes, they concurrently disrupt critical iron-dependent complexes in mitochondrial energy production systems. This collateral interference with electron transport mechanisms impairs cellular energy generation, paradoxically accelerating deterioration in physiologically active tissues despite oxidative stabilization [132]. The resulting metabolic trade-off—achieving pigment preservation while compromising energy homeostasis—reveals fundamental limitations in conventional chelation methodologies. Such antagonistic effects underscore the necessity for intervention strategies that achieve molecular specificity, particularly in decoupling antioxidant effects from essential cofactor depletion through temporally or spatially controlled chelation approaches [133].

Emerging plant-derived alternatives demonstrate selective chelation via ortho-dihydroxyphenyl moieties, binding Cu^2^^+^ while sparing essential Mg^2^^+^ and Ca^2^^+^ ions critical for texture preservation [134]. Lactoferrin, though promising for its dual antimicrobial–chelation capacity, faces scalability challenges due to its pH-dependent iron release, which risks pro-oxidant effects in low-acid vegetables [135]. The field now confronts a trilemma: optimizing chelator specificity to avoid nutrient stripping, balancing redox homeostasis, and maintaining cost-effectiveness. Future innovation must prioritize metabolically intelligent designs: CRISPR-edited vegetables with endogenous metal-binding peptides, or light-responsive chelators activated only during storage stress. Such systems could transcend the current trade-offs, enabling dynamic metal regulation aligned with vegetable physiology rather than static inhibition.

Recent innovations in the application of chemical methods to inhibit vegetable browning are presented in Table 2.

### 3.3. Natural Anti-Browning Method

The shift toward natural anti-browning agents reflects both consumer demand for clean-label products and scientific recognition of their multifunctional mechanisms. While plant-derived inhibitors like seabuckthorn extract and CGA demonstrate competitive binding to PPO active sites via hydrophobic interactions, their industrial adoption faces unresolved challenges [155]. Molecular docking studies showed that quercetin forms a stable π-π stack with amino acid residues of PPO. The number of interacting forces increases slightly at acidic pH, reversibly binding to PPO molecules and decreasing PPO activity [156]. However, batch-to-batch variability in phenolic content due to extraction methods undermines reproducibility—a critical barrier for standardization. Similarly, Similarly, the anti-browning efficacy of the inhibitor in foods decreases at pH values greater than 6.0 due to the ionization of its moiety, which weakens the interaction with the PPO amino acid residues [157]. These pH-dependent limitations highlight the need for microenvironment-tailored formulations [158]. Bioactive peptides exhibited broad-spectrum enzyme inhibition by disrupting FAD cofactor binding in peroxidases [159]. Yet, However, they are susceptible to hydrolysis during storage and are susceptible to environmental influences, so they need to be encapsulated for sustained release [160].

Synergistic combinations, such as 4-hexylresorcinol (4-HR) with ascorbic acid, demonstrate additive PPO inhibition (85% vs. 55% alone) by simultaneously targeting the enzyme’s oxy (4-HR) and deoxy (CGA) states [161,162]. However, natural extracts face an organoleptic trade-off: tannins are potent inhibitors of PPO, but excessive additions produce astringent flavors (tannin content > 2.5 mg/g) that exceed the threshold of consumer acceptance [163]. Future research must prioritize precision synergy—AI-driven optimization of inhibitor ratios to balance efficacy and palatability—while addressing extraction scalability. The path forward demands systems-level approaches integrating multi-omics (e.g., metabolomics-guided inhibitor screening), advanced delivery systems (light-responsive nanocapsules), and circular economy models (upcycled agricultural byproducts). Standardization frameworks must reconcile the inherent variability of natural extracts through robust quantification of active biomarkers (e.g., HPLC-MS for swertiamarin), ensuring batch consistency without compromising cost-effectiveness. Only by addressing these multidimensional challenges can natural anti-browning agents transition from laboratory curiosities to scalable, sensorially neutral solutions for the fresh-cut industry. Although the browning inhibition rate of certain natural extracts is generally lower than that of chemical preservatives, their Generally Recognized as Safe (GRAS) status and absence of synthetic residues align more closely with the clean-label movement and consumer demand for natural ingredients [164]. In contrast, while physical treatments such as heat or irradiation avoid chemical residues, they often exhibit limited efficacy in browning control and may compromise textural integrity-particularly in fresh-cut produce. Therefore, natural techniques offer a balanced alternative, prioritizing safety and sustainability, with performance potentially enhanced through formulation optimization or synergistic applications.

Table 3 summarized recent research exploring the use of natural extracts and bioactive compounds as anti-browning agents, highlighting the effectiveness and versatility of natural methods in preserving color and quality in fresh-cut vegetables. Future research can focus on optimizing the extraction and application of these natural inhibitors to improve their stability and effectiveness in commercial food processing.

## 4. Conclusions and Prospects

The preservation of color in vegetables remains a significant scientific and technological challenge requiring ongoing innovation. Color critically influences consumer perceptions of quality, encompassing not only appearance but also freshness, safety, and nutritional value. This review has summarized current research on color protection technologies, focusing on mechanisms of color degradation such as enzymatic browning, non-enzymatic browning, and chlorophyll degradation. By evaluating physical, chemical, and natural preservation approaches, we have highlighted their respective strengths and limitations, underscoring the need for further research to resolve key gaps.

### 4.1. Current Research and Gaps in Understanding Mechanisms

While advances in physical methods (e.g., modified atmosphere packaging), chemical agents (e.g., anti-browning compounds), and natural extracts (e.g., organic acids, plant antioxidants) show promise, the detailed cellular and molecular interactions of these agents with vegetable tissues remain poorly understood. Most studies focus on efficacy without exploring how these treatments affect enzyme activity, cellular integrity, or membrane permeability across diverse vegetable types. Furthermore, the impact of preservation methods on the nutritional quality—especially antioxidants, vitamins, and phenolic compounds—is insufficiently studied [186]. Deeper mechanistic insights are critical to optimize treatments that balance color retention with nutrient preservation.

### 4.2. Outlook on Novel Anti-Browning Agents and Natural Preservatives

Natural anti-browning agents derived from plant extracts such as rosemary, sage, and green tea offer safer, consumer-friendly alternatives to synthetic chemicals [187]. However, optimizing their dosage, effectiveness, and interactions with other food components requires further research. Exploring underutilized plants and agricultural by-products may reveal sustainable and economically viable new sources of effective color-preserving compounds.

### 4.3. The Promise of Biotechnology: Gene Editing and Plant Breeding

Biotechnological approaches like CRISPR-Cas9 gene editing and selective breeding hold great potential to develop vegetable varieties with inherent resistance to browning and chlorophyll degradation. Targeting genes responsible for PPO activity or enhancing natural antioxidant levels could reduce reliance on post-harvest treatments and extend shelf-life intrinsically [188]. However, regulatory, ethical, and consumer acceptance challenges must be addressed through transparent labeling and education to facilitate adoption.

### 4.4. Towards Integrated, Holistic Preservation Systems

Given the complexity of color preservation, integrated strategies combining physical, chemical, natural, and biotechnological methods are likely to be most effective. Advances in intelligent packaging—embedding sensors that detect spoilage and trigger-controlled release of anti-browning agents—offer promising, resource-efficient solutions that minimize preservative use and prolong shelf-life sustainably.

### 4.5. The Importance of Cross-Disciplinary Collaboration and Future Research Directions

Achieving breakthroughs in color preservation demands collaboration across food chemistry, plant science, biotechnology, and materials science. Integrating diverse expertise can lead to novel, practical solutions such as breeding vegetables optimized for post-harvest stability and developing advanced packaging tailored to perishable produce. Future research should also prioritize consumer preferences, safety, sustainability, and transparent communication to ensure public trust and acceptance of new technologies, especially those involving genetic modification or novel additives.

In summary, the future of color protection for vegetables is bright, with significant potential for enhancing both the shelf-life and nutritional value of fresh produce. By pursuing a balanced approach that combines scientific rigor with practical application, the food industry can meet consumer demands for high-quality, nutritious, and visually appealing products while addressing the global need for sustainable food systems. As research progresses, the development of integrated color-preservation systems that harness the strengths of multiple preservation technologies will be crucial, paving the way for a new era of innovation in vegetable quality preservation. From an application standpoint, several of the strategies exhibit promising translational potential. However, challenges remain in terms of production cost, shelf stability, and large-scale implementation. Future research should focus not only on mechanistic refinement but also on optimizing these technologies for commercial scalability.

## Figures and Tables

**Table 1:** Recent innovations in physical methods for delaying vegetable browning.

Methods		Applications	Conclusions	References
Cold treatment	Orthogonal ultrasound-assisted freezing	Potato	Sample tissue structure integrity, and significant increase in phenolic content	[47]
	Static magnetic fields freezing	Potato	SMF does not significantly affect enzymatic browning	[82]
	Isometric freezing	Spinach	Thawed samples had similar cellular integrity and lysis as fresh spinach	[49]
		Potato	The total phenolic content and antioxidant capacity of the samples increased and browning was delayed by 1 week	[51]
	Liquid nitrogen spray freezing	Chinese matrimony vine	PPO activity was significantly reduced	[83]
Heat treatment	High-humidity hot air impingement blanching	Chili peppers	Reduce the residual activity (0.52%) and Browning index (7.09) of POD	[57]
	Hot air-assisted radio-frequency heating	Red bell pepper	Higher ascorbic acid retention and free radical scavenging activity	[84]
	Infrared hot bleaching	Mushroom	IRHAD provides samples with the highest oxidation resistance and the lowest total color difference.	[85]
	Catalytic infrared heating coupled with holding time (CIRH)	Mushroom	CIRH enhanced the maintenance of microstructure and increased the retention of total phenols	[86]
	Microwave ironing and bleaching	Asparagus	The highest free radical scavenging capacity and total phenol content were found in samples blanched at 300 W for 4 min	[87]
	Vacuum-assisted blanching	Zucchini slice	Less color difference with hot water rinsing, TFC reduced to 39.91%.	[88]
	Infrared and convection drying	Lotus root	Inhibits the activity of PPO and POD enzymes	[89]
	Thermosonication blanching	Carrot	22/40 kHz downregulated POD genes expression	[90]
		Carrot	Thermal sonication at 60 °C significantly increased the hardness of the sample tissue.	[91]
		Lotus root	TS decreased the browning degree, POD activity and MDA content	[92]
	Catalytic infrared heating	Carrot	90% inactivation of POD enzyme activity by short-term CIR blanching	[93]
	Infrared hot air drying	Garlic	IRHAD retains nutrients and antioxidant activity.	[94]
		Bitter Gourd	BPS-I exhibits enhanced antioxidant activity in vitro	[95]
	Ultrasound infrared drying	Carrot	US-IR drying does not adversely affect the color of the sample	[96]
	Microwave heat	Potato	1.1 kW microwave heating can reduce the anti-nutrient load of potato	[97]
	Electromagnetic field-assisted blanching	Cabbage	The dielectric properties of blanched cabbage were significantly improved and color retention was increased	[98]
	Osmosonication drying	Ginger	Significantly improves the efficiency of enzyme inactivation	[99]
Gas-conditioning treatment	Map	Cauliflower	6.06% O_2_; 11.43% CO_2_ can inhibit browning most effectively	[100]
		Lily	10% O_2_, 5% CO_2_, 85% N_2_ inhibits the synthesis and accumulation of phenolics, as well as oxidative reactions	[101]
		Amaranth	10% O_2_, 10% CO_2_ and 80% N_2_ are effective in maintaining chlorophyll and ascorbic acid content and antioxidant enzyme activities	[102]
		Cucumber	2% O_2_, 7% CO_2_, and 91% N_2_ reduced the ability of bacterial biofilm formation	[103]
		Bell pepper	10% O_2_; 45% CO_2_ slows down metabolism	[104]
		Daylily bud yield	HRW treatment maintained redox balance by inhibiting O_2_^•−^ and H_2_O_2_ accumulation	[105]
	Microporous MAP	Bean pods	MPPP12 (14% O_2_ and 4% CO_2_) effectively preserved chlorophyll content	[106]
		Mulberry leaf lettuce	Modified polyethylene packaging reduced the respiration intensity of the samples, and the inhibition intensity was positively correlated with the enzyme activity inhibition rate	[107]
	Ethanol fumigation MAP	Potato	Effectively prevents the conversion of branched-chain starch to chloroplasts	[108]
High-pressure treatment	High pressure inert gases	Potato	4 MPa high pressure effectively inhibited respiration rate and biofilm oxidation	[71]
Irradiation treatment	Diode laser irradiation (450 nm)	Potato	Inhibition of PPO and POD expression and enhancement of antioxidant activity	[75]
	He-Ne laser irradiation	Shepherd’s purse Cauliflower Turnip	Enhanced antioxidant, antibacterial, anti-inflammatory, and anticancer activities	[109]
	UV-B	Spinach	High-dose UV-B irradiation reduced leaf yellowing	[110]
	UV-C	Carrot	Increased total phenolic content and antioxidant capacity	[111]
	Intensive pulsed light	Mushroom	LPL altered the secondary and tertiary structure of PPO leading to inactivation	[79]
		Shiitake mushroom	After 25 pulses of IPL energy of 400 J, the PPO activity was reduced by 42.83%	[112]
		Carrot juice	The production of hydroxyl radicals accelerates the death of bacteria	[113]
Other methods	Pulsed magnetic field	Cucumber Juice Lettuce Juice Carrot Juice Tomato Juice	Maintain the color and flavor of vegetable juice while controlling pathogenic bacteria	[114]
	Tri-frequency ultrasound	Carrot	POD activity decreased by 81.43%	[115]
	Osmosonication	Ginger	The highest phenol content and antioxidant activity were obtained at 50 kHz	[116]
	Ultrasound	Coffee leaf	Ultrasonic regulation of phenolic metabolism	[117]
	Pulsed electric field and ultra-sonication	Spinach juice	The inactivation rates of POD and PPO increased from 0.85 and 0.025 Abs/min to 0.18 and 0.011 Abs/min	[118]
	Electrospray	Cabbage juice	The secondary and tertiary structures of PPO and POD are destroyed	[119]
	Multi-frequency ultrasound	Chinese cabbage	Dual-frequency ultrasound showed more positive sensory properties	[120]

**Table 2:** Recent innovations in chemical methods for delaying vegetable browning.

Method		Applications	Conclusions	References
Acidifying agents	Acetic acid	Potatoes	Delayed ascorbic acid degradation and enhanced cell wall integrity	[121]
	Oxalic acid	Lotus Root	Inhibited H_2_O_2_ and O^2−^ production and reduced POD and PPO activities	[136]
	Citric acid	Lotus root	Reduced production of H_2_O_2_ and O^2−^ while decreasing malondialdehyde, PPO and POD levels	[137]
Antioxidants	Citric acid	Cabbage	Reduced residual PPO activity	[138]
	Glutathione	Lotus root	Increasing PAL activity to stimulate total phenol accumulation while inhibiting PPO and POD activity	[139]
		Flammulina velutipes	Increase soluble solids content	[140]
	L-arginine L-cysteine L-methionine	Broccoli	Reduced metabolism by inhibiting endogenous ethylene production led to a lower browning rate	[141]
	L-cysteine	Lotus root	Conversion of phenolic compounds to cysteine adducts as competitive inhibitors of PPO	[142]
		Flammulina velutipes	Regulates ROS metabolism and stimulates endogenous H_2_S production	[143]
	Melatonin	Sweet potato	Induces the expression of genes related to antioxidant pathway and reduces enzyme activity, ROS content and membrane lipid peroxidation	[91]
		Mushroom	100 μM melatonin decreased PPO gene expression and enzyme activity	[144]
		Lotus seed	Inhibited oxidase activity and increased endogenous MT	[145]
		Spinach	0.20 mg/mL melatonin retarded chlorophyll degradation but increased POD activity	[146]
		Lotus seeds	Reduction in membrane oxidative damage in mitochondria by stimulating antioxidant enzymes to scavenge ROS	[147]
		Broccoli	Endogenous melatonin homeostasis led to downregulation of the expression of chlorophyll degradation-related and ethylene synthesis-related genes	[148]
	Naclo	Cabbage	The synergistic effect of multi-frequency ultrasound in a sweeping mode combined with naclo is effective in sterilization	[149]
Chelating agents	Oxalic acid	Lotus root	Inhibition of H_2_O_2_ and O^2−^ production and reduction in POD and PPO activity	[136]
	Kojic acid derivatives	Potato	Competitive inhibitor of tyrosinase (IC_50_ = 3.23 ± 0.26 μM)	[150]
	S-Furfuryl thioacetate	Potato Eggplant Lettuce	Changing the conformation of PPO by chelating Cu^2+^	[151]
		Potato	Decreased PPO activity by chelating Cu^2+^ and acting on the residues of PPO	[152]
	Kojic acid-1,3,4-oxadiazole derivatives	Mushrooms	Binds to Cu^2+^ in the active region, altering the secondary structure of tyrosinase	[153]
	Fern 6’-O-azelate	Potato	The inhibition of monophenolase was 31.4 ± 1.36%	[154]

**Table 3:** Recent innovations in natural anti-browning method for delaying vegetable browning.

Natural Additives	Applications	Conclusions	References
Γ-aminobutyric acid	Stem lettuce	Inhibition of PPO activity by delaying the expression of lsppo	[7]
Sea buckthorn leaf extract	Potato	Competitive inhibitor of PPO (IC_50_ = 0.7 mg/mL)	[155]
Chlorogenic acid	Potato	Rearrangement of PPO secondary structure by hydrophobic interaction	[158]
4-Hydroxycinnamic acid		Interaction with PPO through hydrogen bonding and hydrophobic interactions converted α-helix into β-sheet	[156]
Cod peptide	Potato	High concentration of Cod peptide decreased the total phenol content, PPO, POD and PAL activities	[159]
Mulberry root bark 2-arylbenzofuran derivatives		The active site interacted with Cu^2+^ and peroxide ions to enhance antioxidant activity and inhibit tyrosinase activity	[165]
Pineapple extract Onion extract Pepper extract Honey	Sweet potato	All of them inhibited PPO, among which honey had the highest inhibition rate of 41.39–48.0%	[166]
Purslane extract	Potato	Ultrasound-coupled extract treatment is more effective in maintaining cell membrane integrity, inhibiting PPO and POD activities, and improving antioxidant capacity	[167]
Golden ginkgo tannins	Lotus root	Acted as a reversible mixed competitive inhibitor of tyrosinase (IC_50_ = 123.90 ± 0.140 μg/mL)	[168]
Beef oregano extract	Mushroom	The greatest inhibitory effect was observed on PPO with 64.50% inhibition	[169]
Citronella hydrosol Rose hydrosol	Taro	Terpenoids were effective in reducing PAL, POD and PPO activities	[170]
Mango peel extract	Mashed potato	Competitive inhibition of PPO (IC_50_ = 0.3 mg/mL)	[171]
Probiotics fermentation suspension	Lotus root	Decreased activities of PPO, POD and PAL, reduced TPC and soluble quinones	[172]
	Lotus root	Slowed down physiological responses and inhibits enzymatic browning-related enzyme activities	[173]
Eucalyptus citriodora essential oil	Cabbage	Essential oil-based Pickering emulsion maintained color, chlorophyll content	[174]
Bacteriophages	Lettuce, Cucumber Carrot	Improvement of organoleptic quality of vegetables by sterilization (Disrupts the cellular structural properties of bacteria)	[175]
Litseacubeba essential oil	Cucumber juice Carrot juice Spinach juice	The optimal synergistic effects were found using PMF (3 times under 8 T, 60 pulses) treatments combined with 1.5 mg/ml	[176]
Basil essential oil	Cabbage	Ultrasound and basil essential oil nanoemulsion disrupted the increase in intracellular ROS and extracellular MDA leading to bacterial sterilization	[177]
Litsea cubeba essential oil	Bitter gourd Juice Carrot juice Cucumber juice Spinach juice	Inhibit bacterial respiratory tract metabolism, hinder bacterial nucleic acid replication	[178]
Citral	Carrot	US and CLON reduced the amount of Sh. Flexneri adhering to the sample surface while retaining important quality attributes	[179]
Baobab seed oil	Mushroom	Not only improved the freshness of mushrooms but also maintained the structural stability	[180]
Clove essential oil	Mushroom	Ultrasound-assisted clove essential oil nanoemulsions effectively maintained the quality characteristics of mushroom	[181]
	Cucumber Lettuce	Enhances vegetable color by killing bacteria	[182] [183]
Cumin	Cucumber juice Tomato juice	Cold nitrogen plasma-modified cumin aldehyde/β-cyclodextrin inclusion complexes for effective color retention in vegetable juices	[184]
Bacillus velezensis	Eggplant	The activity of ROS scavenging enzyme was enhanced and the antioxidant capacity was improved	[185]

## Data Availability

No new data were created or analyzed in this study. Data sharing is not applicable to this article.

## Supplementary Materials

The following supporting information can be downloaded at: https://www.mdpi.com/article/10.3390/foods14132222/s1, Figure S1. Schematic of isochoric chamber and generalized freezing process; Figure S2. Heat transfer mechanism depiction in HHAIB; Figure S3. Functional mechanism of gas transfer phenom.

## Abbreviations

PPO	polyphenol oxidase
MAP	modified atmosphere packaging
HHAIB	high-humidity hot air impingement blanching
MAB	microwave-assisted blanching
PEF	pulsed electric fields
HPP	High-pressure processing
4-HR	4-hexylresorcinol
