# Supplementary material for: Mechanistic Insights into Vegetable Color Stability: Discoloration Pathways and Emerging Protective Strategies
Source: Foods. 2025 Jun 24;14(13):2222. doi: 10.3390/foods14132222 (PMC12248490; doi:10.3390/foods14132222)
Supplement: Supplementary file 1 [file foods-14-02222-s001.zip › foods-3700524-supplementary.pdf]

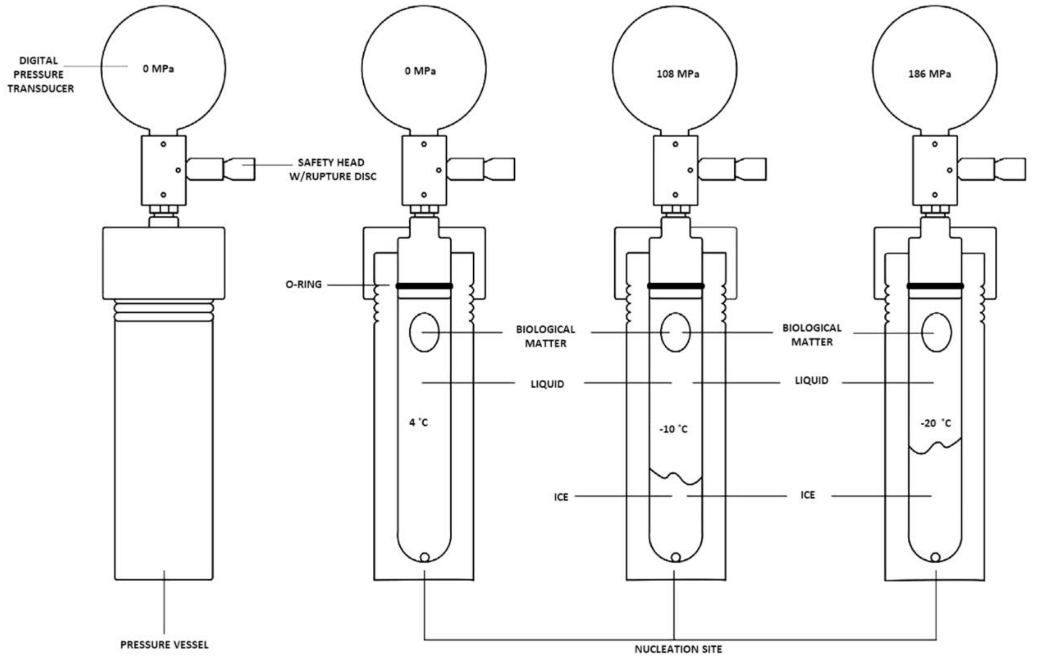

Figure S1. Schematic of isochoric chamber and generalized freezing process.

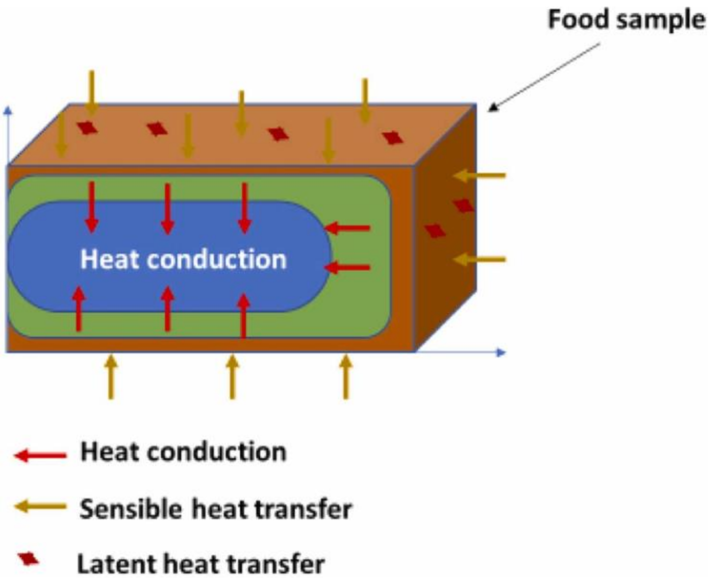

Figure S2. Heat transfer mechanism depiction in HHAIB.

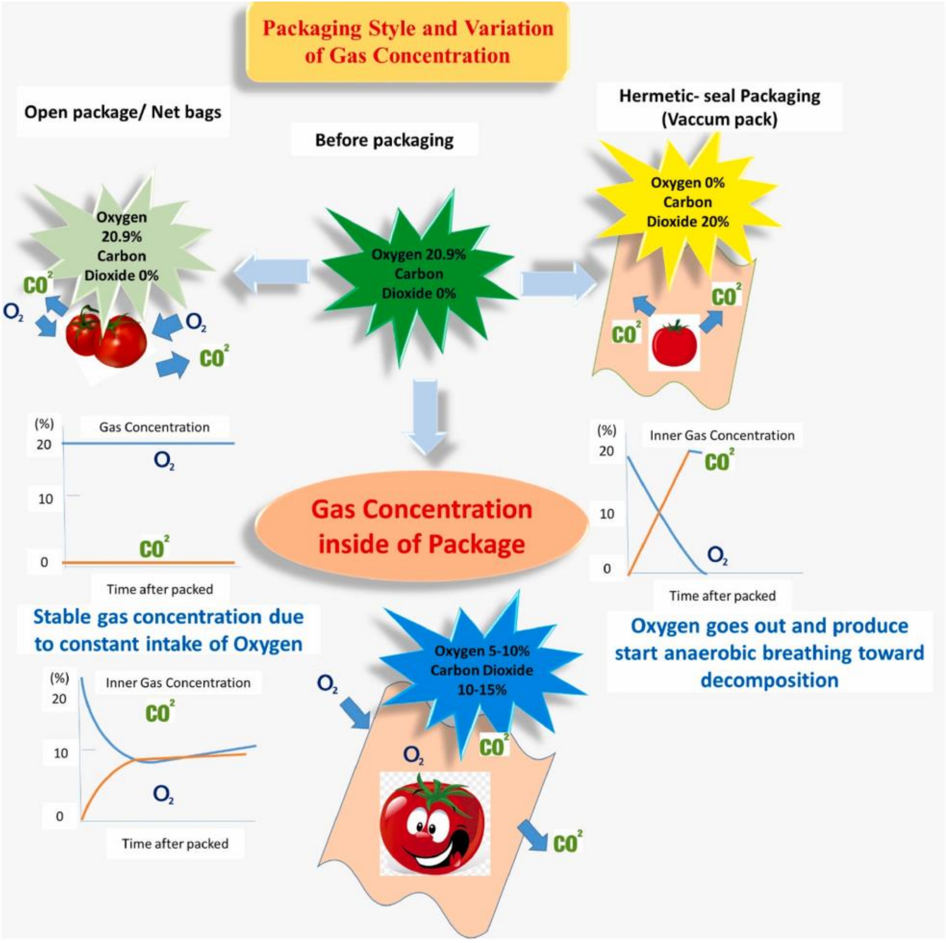

Figure S3. Functional mechanism of gas transfer phenomenon.
